# Brisket Disease Is Associated with Lower Volatile Fatty Acid Production and Altered Rumen Microbiome in Holstein Heifers

**DOI:** 10.3390/ani10091712

**Published:** 2020-09-22

**Authors:** Naren Gaowa, Kevin Panke-Buisse, Shuxiang Wang, Haibo Wang, Zhijun Cao, Yajing Wang, Kun Yao, Shengli Li

**Affiliations:** 1 State Key Laboratory of Animal Nutrition, Beijing Engineering Technology Research Center of Raw Milk Quality and Safety Control, College of Animal Science and Technology, China Agricultural University, Beijing 100193, China; narengaowa@cau.edu.cn (N.G.); harper.wang@cau.edu.cn (H.W.); caozhijun@cau.edu.cn (Z.C.); yajingwang_cau@163.com (Y.W.); 2 USDA Agricultural Research Service, US Dairy Forage Research Center, Madison, WI 53706, USA; kevin.panke-buisse@usda.gov; 3 Laboratory of Animal Nutrition, Institute of Animal Science, Academy of Animal Science and Veterinary Medicine, Qinghai University, Xining 810016, China; 15850594890@163.com; 4 College of Animal Science, Xinjiang Agricultural University, Urumqi 830052, China; cauyaokun@126.com

**Keywords:** brisket disease, rumen microbiome, Holstein heifer, pulmonary artery pressure, bovine pulmonary hypertension

## Abstract

**Simple Summary:**

Development of the dairy industry in the high-altitude plateau environment through incorporation of Holstein cows is complicated by the risk of brisket disease. While the physiological effects of brisket disease are well-studied, its effects on rumen function and microbial community composition are not. There are clear shifts in volatile fatty acids production and rumen microbial community composition in Holstein heifers suffering from brisket disease. Observed shifts reveal key genera associated with healthy and disease states and suggest that bovine brisket disease is associated with impaired rumen functioning. This work supports further understanding of the roles of key rumen taxa in bovine brisket disease, with particular focus on candidate rumen biomarkers in healthy animals that may be able to reduce economic losses for farmers.

**Abstract:**

Brisket disease is heritable but is also associated with non-genetic risk factors and effects of the disease on the rumen microbiome are unknown. Ten Holstein heifers were exposed to the plateau environment for three months and divided into two groups according to the index of brisket disease, the mean pulmonary arterial pressure (mPAP): brisket disease group (BD, n = 5, mPAP > 63 mmHg) and healthy heifer group (HH, n = 5, mPAP < 41 mmHg). Rumen fluid was collected for analysis of the concentrations of volatile fatty acids (VFAs). Extracted DNA from rumen contents was analyzed using Illumina MiSeq 16S rRNA sequencing technology. The concentration of total VFA and alpha-diversity metrics were significantly lower in BD group (*p* < 0.05). *Ruminococcus* and *Treponema* were significantly decreased in BD heifers (*p* < 0.05). Correlation analysis indicated that 10 genera were related to the mPAP (*p* < 0.05). Genera of *Anaerofustis, Campylobacter,* and *Catonella* were negatively correlated with total VFA and acetic acid (R < −0.7, *p* < 0.05), while genera of *Blautia, YRC22, Ruminococcus,* and *Treponema* were positively related to total VFA and acetic acid (R > 0.7; *p* < 0.05). Our findings may be a useful biomarker in future brisket disease work.

## 1. Introduction

The process of producing more food while decreasing environmental impact has become a global challenge and requires what has been referred to as the “sustainable intensification” of global agricultural production [1]. High altitude environments (1500–3500 m) can create physiological challenges due to the low atmospheric pressure and oxygen availability [2]. Although the Yak (*B. grunniens*) and Tibetan cattle (*B. grunniens × B. primigenius Taurus*) have been adapted to high altitude and low oxygen environments, their production cycle is long and milk production is low [3]. Thus, the introduction of Holstein cows to the Tibetan plateau could be an effective way to alleviate the shortage of milk. Unfortunately, bovine brisket disease (BD), which is initiated by high altitude pulmonary hypertension (HAPH), could affect 3–25% of cattle transported from low to high altitudes and cause financial losses to farmers [4,5].

Acute altitude exposure results in a marked reduction of arterial oxygen saturation and oxygen supply to the cardiovascular system [6], and increases mean pulmonary arterial pressure (mPAP) in un-adapted individuals. Heifers had different adaptability to the plateau environment when transported from low to high altitudes. Heifers suffering from the HAPH are characterized by thickened pulmonary artery adventitia, thinner pulmonary artery intima, and higher vascular media area percentage [7]. However, effects on the gastrointestinal tract of cattle suffering from bovine brisket disease are unknown.

The rumen plays an important role in providing necessary nutrients to the animal [8] and has several important physiological functions, including the absorption of volatile fatty acids (VFAs) [9], nutrient transport [10], and metabolic activity and protection [11]. VFAs are crucial to the maintenance, growth, and production performance of ruminants [12]. Many studies have found that VFAs are related to the rumen microbiome [13,14,15]. Tong et al. found that *Bacteroides, Ruminococcus 2,* and *Candidatus Saccharimonas* were positively correlated with ruminal propionate proportion [14]. Ishaq et al. indicated that Firmicutes were negatively correlated with total VFA, total acetate, and total propionate [15]. However, the composition of the ruminal microbial community is influenced by several factors, such as age, diet, health status, host species, geographical location, and whether the host has received antibiotic treatment [16]. High-altitude environments can impair rumen fermentation and elevate the basal metabolic rate of Holstein cows [17]. Moreover, the plateau environment may also cause changes in the abundance and composition of the rat gut microbiome [18]. The aim of this study was to identify potential changes in rumen VFAs and bacterial community composition between healthy heifers and heifers with bovine BD.

## 2. Materials and Methods

### 2.1. Ethics Statement

This experiment was approved by Institutional Animal Care and Use Committee at China Agricultural University (Beijing, P. R. China; permit no. AW10102020-1-1). All animals involved in this study were housed in the same pen and had free access to water throughout the experiment.

### 2.2. Experimental Design and Sample Collection

In this study, 2000 Holstein heifers were transported from Xian (Shaanxi province, China 1027 m altitude) to Lhasa (Tibet, China 3658 m altitude) in March 2016 and exposed to the plateau environment for three months to adapt. Details on feeding are shown in Appendix A. The heifers were housed in the same pen and had free access to water. They were fed three times daily, and the total mixed ration was offered ad libitum to yield 5% feed refusals. Ten of them (16–18 months old, non-pregnant, 495 ± 15 kg) were divided into two groups according to the mean pulmonary arterial pressure [4]: brisket disease group (BD, n = 5, mPAP higher than 63 mmHg) and healthy heifer group (HH, n = 5, mPAP lower than 41 mmHg). Clinical signs, including labored breathing, droopy ears, distended external jugular veins, and the edema of brisket and underjaw, were used to find the heifers with brisket disease before measuring mPAP.

The mPAP, blood oxygen saturation, and breathing rate were measured three hours after feeding in the morning. To determine mPAP, the right external jugular vein was pricked using a needle, and a Swan-Ganz catheter (7F) was inserted. A three-way stop cock was used to connect a pressure transducer (Millar Instruments, Houston, TX, USA) to a physiological recorder (Powerlab ML786), and the pressure wave was viewed using Chart5 computer software (AD Instruments, Colorado Springs, CO, USA) to determine mPAP. The transducer was placed at the same level as the heart and the catheter was guided into the right ventricle and pulmonary artery to record the pressure after calibrating to the baseline, as previously described [4]. Measurements were repeated three times and the average of 20 pressure cycles was used to calculate the pressure value. After measuring mPAP, blood oxygen saturation was measured using the method described by Michaux et al. [19]. Under calm conditions, the breathing rates were counted by two veterinarians at the same time. They stood on the left and right sides of the cow’s tail while simultaneously observing the fluctuation of the abdominal rise and fall during breathing. One rise and fall as one breath was counted. The number of breaths per minute was recorded as one measurement. Each measurement was taken three times in a row and the average of the three measurements was used as the breathing rate. 

Selected heifers were sacrificed at 1 pm after all the measurements above. Rumen was shaken before cardia cutting. Rumen contents were poured into the 1 L sterilized beaker, then divided into 4 tubes (2 mL) immediately. Tubes were flash frozen in liquid nitrogen, then stored at −80 °C for further bacteria analysis. Rumen liquid were obtained by squeezing the rumen contents through four layers of sterile cheesecloth, then stored in 10 mL tubes at −20 °C for further VFAs analysis. The concentrations of VFAs in rumen fluid were determined using a gas chromatograph (6890N; Agilent technologies, Avondale, PA, USA) equipped with a capillary column (HP-INNOWax 19091N-213, Agilent). Details followed the description in co-author’s report [20].

### 2.3. DNA Extraction, 16S rRNA Gene Amplicon Preparation and Sequencing

For DNA extraction, 1 g of mixed rumen contents was divided from the raw samples. DNAs were extracted using Qiagen’s DNA Extraction Kit™ (Qiagen, Hilden, Germany) following the manufacturer’s instructions. The qualities of the DNA were appraised using a NanoDrop ND-1000 Spectrophotometer (NanoDrop Technologies, Wilmington, DE, USA). The extracted DNA was amplified by PCR with the KAPA HiFi Hotstart ReadyMix PCR kit (Carlsbad Life Technologies, USA). The V3–V4 region of the bacterial 16S rRNA gene was amplified using primers F341 (5’-ACTCCTACGGGRSGCAGCAG-3’) and R806 (5’-GGACTACVVGGGTATCTAATC-3’) [21]. The PCR products were gathered from 2% agarose gels and purified with a QIAquick PCR Purification Kit (Qiagen, Hilden, Germany). Purified DNAs were re-quantified using an Agilent DNA 1000 Kit (Agilent Technologies, Waldbronn, Germany). Library quality was assessed on a Qubit 2.0 Fluorometer (Life technologies, Grand Island, NY, USA). Sequencing library preparation was done using NEBNext ultra DNA sample preparation kit (NEB, USA) following the manufacturer’s protocol. Then, reads of approximately 250–300 bp paired-end were sequenced on the Illumina MiSeq platform. 

### 2.4. Bioinformatics and Statistical Analyses

The quality control of raw data was done by FastQC (https://www.bioinformatics.babraham.ac.uk/projects/fastqc/). Concatenated sequences were detected using USEARCH (http://www.drive5.com/usearch/). Sequences analyses and alpha diversity were performed using QIIME pipeline (version 1.5.0 as shown in the reference) [22]. Beta diversity was measured according to weighted UniFrac distances and displayed using principal coordinate analysis (PCoA) based on R package ‘vegan’ (https://cran.r-project.org/web/packages/vegan/). The LEfSe analysis followed the steps online (https://huttenhower.sph.harvard.edu/galaxy) to find differentially expressed biomarkers with *p*-value < 0.05 and linear discriminant analysis (LDA) score > 3.

The results were expressed as means ± standard deviations. Data were analyzed using independent samples t-test with the SAS 9.0 (SAS Inst. Inc., Cary, NC, USA). Significant differences were determined based on *p* < 0.05. Pearson correlation were created using the “cor” function in R (https://cran.r-project.org/bin/windows/base/old/3.4.0/) the statistical computing and graphics software, with default parameters and the ‘corrplot’ package using data among genera, VFAs, blood oxygen saturation (BOS), average breathing rate (ABR), and mPAP in each heifer.

The 16S rRNA raw reads obtained from rumen content were submitted to NCBI with project accession number of PRJNA598286. 

## 3. Results

### 3.1. Mean Pulmonary Arterial Pressure, Blood Oxygen Saturation Breathing Rate and Rumen VFAs of the BD and Healthy Heifer Group (HH) Animals

The mPAP was significantly higher in the BD group compared with the HH group (Table 1, *p* < 0.01). Blood oxygen saturation and average breathing rate were lower in the BD group compared with the HH group (*p* < 0.05). The concentration of total rumen VFAs in the HH group was significantly higher than that in the BD group (Table 2, *p* < 0.05). Although there were no significant differences in VFA profiles, healthy heifers tended to have a numerically higher proportion of acetic acid (*p* = 0.0758) than the BD group.

### 3.2. Changes of Rumen Predominant Microbiota

**Figure 1:**
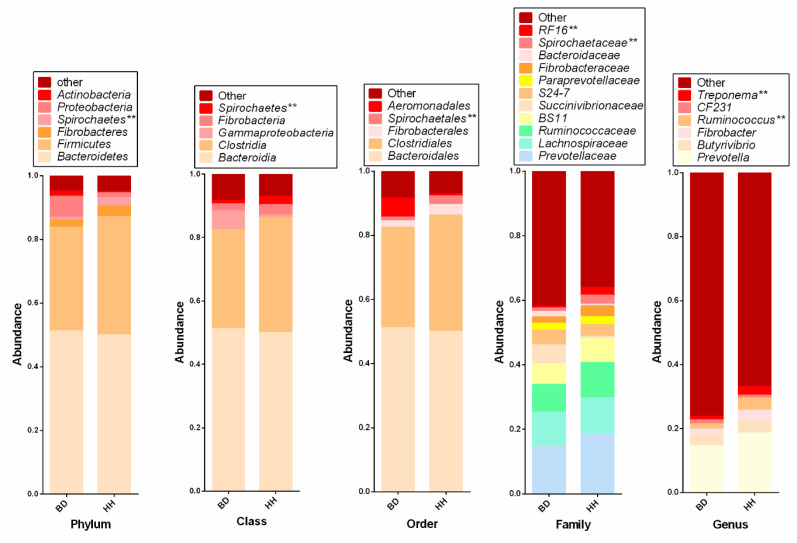
Distribution of predominant bacteria (abundance > 1%) in rumen content. BD = brisket disease group, n = 5; HH = healthy heifer group, n = 5; **: *p* < 0.05.

As shown in Figure 1, six dominant bacterial phyla were measured (abundance > 1%), with the two predominant phyla being *Bacteroidetes* and *Firmicutes*, accounting for 50.6% and 34.9% of the total sequences, respectively. Bacteria from the class *Spirochaetes* and the order *Spirochaetales* were more abundant in HH than in BD (*p* < 0.05). The predominant bacterial family *RF16* (*p* < 0.05) and *Spirochaetaceae* (*p* < 0.05) were lower in BD compared to HH (0.5% vs. 2.3% and 1.1% vs. 2.7%, respectively). The genera *Ruminococcus* (*p* < 0.05) and *Treponema* (*p* < 0.05) constituted 3.9% and 2.6% in HH, whereas only 1.5% and 1.1% in BD, respectively.

### 3.3. Alpha and Beta Diversity of the Rumen Microbial Community

**Figure 2:**
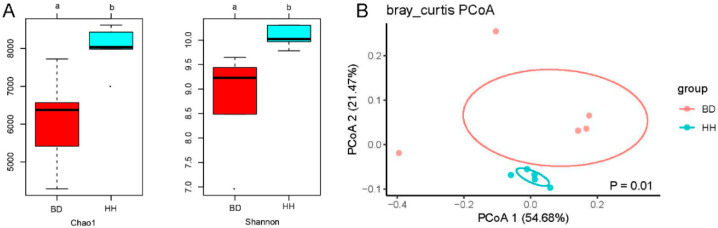
Alpha and beta diversities of the rumen microbial community. (**A**) Bacterial community richness (Chao1) and diversity index (Shannon) of rumen samples of the brisket disease group (BD) and healthy heifer group (HH); significant differences (*p* < 0.05) were shown via using a and b; (**B**) Principal coordinate analysis (PCoA) of bacterial community composition of the ruminal microbiota in BD and HH. PCoA plots were constructed using the weighted UniFrac distance matrix.

A summary of richness and diversity index is presented in Figure 2A. The Chao1 and Shannon index of the BD group was lower than those of the HH group (*p* < 0.05). PCoA of ruminal genera demonstrated clustering of samples between HH and BD groups, PCoA1 and PCoA2 explained 54.68% and 21.47% of the variation in the dataset, respectively (Figure 2B). Thus, alpha and beta diversities in the BD group were distinctly separate from heifers in the HH group.

### 3.4. Microbial Species with Significant Differences

**Figure 3:**
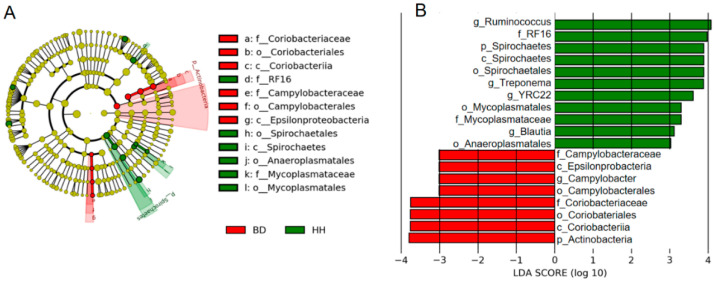
Linear discriminant analysis (LDA) effect size (LEfSe). (**A**) The cladogram diagram shows the microbiota with significant differences in the two groups. Red and green indicate different groups, with the species classification at the level of phylum, class, order, family, and genus shown from the inside to the outside. The red and green nodes in the phylogenetic tree represent microbial species that play an important role in the brisket disease group (BD) and healthy heifer group (HH), respectively. Yellow nodes represent species with no significant difference. (**B**) Microbiota with significant difference that have a LDA score greater than the estimated value; the threshold score is 3.0. The length of the histogram represents the LDA score, which compares the degree of influence of species with significant difference between groups.

The LDA effect size (LEfSe) was performed to reveal the significant ranking of abundant modules. There were significant differences in the community compositions between HH and BD groups. The cladogram showed differences in 19 taxa between HH and BD groups (Figure 3A). The plot from LEfSe analysis displays LDA scores of microbial taxa with significant differences between the two groups (Figure 3B). At the genus level, the relative abundances of *Ruminococcus*, *Treponema*, *YRC22, Blautia,* and *Campylobacter* were significantly different between the HH and BD groups.

### 3.5. Correlation between Genera and Physiological Indicators of Heifers

**Figure 4:**
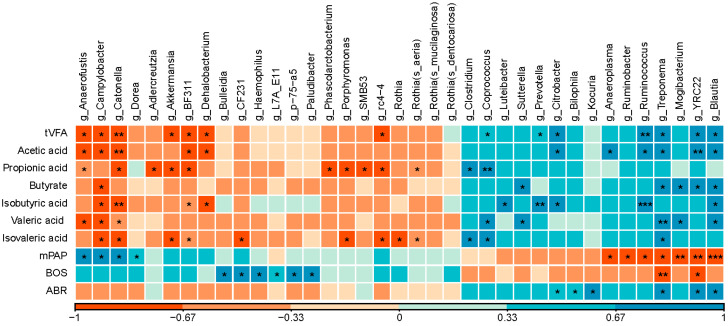
Pearson correlation matrix between genera and the physiological indicators of heifers. The scale of the colors is denoted as follows: the more positive the correlation (closer to 1), the darker the shade of blue; the more negative the correlation (closer to −1), the darker the shade of red. ABR = average breathing rates; mPAP = mean pulmonary arterial pressure; BOS = blood oxygen saturation; tVFA = total volatile fatty acid. *: *p* < 0.05; **: *p* < 0.01; ***: *p* < 0.001.

As shown in Figure 4, 10 genera significantly correlated with pulmonary pressure (R > 0.7 or R < −0.7, *p* < 0.05). Among them, three genera negatively correlated with total volatile fatty acids (tVFA) and acetic acid (*Anaerofustis, Campylobacter*, and *Catonella*; R < −0.7, *p* < 0.05). Genera of *Anaerofustis* and *Campylobacter* were negatively related to valeric acid (R < −0.7, *p* < 0.05). Genera of *Campylobacter* and *Catonella* were negatively related to the concentration of isobutyric acid and isovaleric acid (R < −0.7, *p* < 0.05). Four genera positively correlated with tVFA and acetic acid (*Ruminococcus, Treponema, YRC22,* and *Blautia*; R > 0.7, *p* < 0.05). Genera of *Treponema, YRC22,* and *Blautia* were positively related to butyrate and ABR (R > 0.7, *p* < 0.05). Genera of *Treponema* and *YRC22* negatively associated with BOS (R < −0.7, *p* < 0.05). 

## 4. Discussion

Special geological and climatic environments can cause increased susceptibility to intestinal diseases, and this has been observed for humans and other vertebrates exposed to high altitudes [23]. Insufficient energy aggravates intestinal injury and promotes bacterial and endotoxin translocation under a high-altitude hypoxic environment [24]. Rumen microbiota play a critical role in the gastrointestinal tract by utilizing nutrients within the rumen and providing 70~80% of metabolizable energy for the host [25]. Microbial metabolites produced are mainly VFAs, which are absorbed through the ruminal epithelium. In the current study, lower rumen total VFAs in the BD group compared to the HH group suggest less energy was available for heifers affected by high-altitude-induced brisket disease.

As shown in this study, *Bacteroidetes* and *Firmicutes* were the most predominant phyla in the rumen, as recognized by previous studies, including pre-weaning goat [26], heifer [27], and yak [23,28]. A recent study reported that dietary factors are more influential to the rumen microflora than host species and geographical environment [29]. However, in this study, under the same conditions of diet and geographical environment, the richness and diversity of rumen microbial community in the HH group were higher than in the BD group, which might be due to the health status of the host. In addition, the PCoA showed that BD samples were clearly distinguishable from HH group samples, suggesting distinct microbiomes between healthy and sick heifers. The BD heifers also had clinical signs such as labored breathing, droopy ears, distended external jugular veins, and the edema of brisket and underjaw. This observation may further improve understanding of the role of health status in bacterial community.

In the current study, results of LEfSe illustrated that the distribution difference of rumen bacteria in different fractions can be observed in both HH and BD groups. As the predominant genera in our study, *Ruminococcus* and *Treponema* were significantly lower in BD heifers and positively correlated with tVFA and acetic acid. These findings indicated that decreased *Ruminococcus* and *Treponema* could affect the concentration of tVFA and acetic acid in heifer rumens suffering from high-altitude-induced brisket disease. As many reports have shown, genera *Ruminococcus* and *Treponema* are key contributors of carbohydrate-active enzymes [30], which can break down plant cell walls and cooperatively contribute to dietary cellulose, hemicellulose [31], and pectin deconstruction [32,33]. Additionally, *Ruminococcus* has also been confirmed to be deficient in rats’ gut under a hypobaric hypoxia condition [34]. *Treponema* is relatively abundant in Tibetan Chickens, which can live on the high-altitude plateau, when compared to low-altitude broiler chickens [35]. The genus of *Blautia*, which has been reported as an acetate producing bacteria [36], was significantly different between groups according to the LEfSe results. *Blautia* was more abundant in healthy heifers and positively correlated with the concentration of tVFA, acetate, and butyrate. One study has reported an increased abundance of *Blautia* in healthier people as compared to those in poorer health [37]. At the same time, Barcenilla et al. found that *Blautia* had the ability to synthesize butyric acid and some other short chain fatty acids [38]. The abundance of *Blautia* may influence the concentration of tVFA, acetate, and butyrate in the rumen of heifers with high-altitude induced brisket disease. These results indicate that *Ruminococcus*, *Treponema*, and *Blautia* may be useful biomarkers related to bovine brisket disease.

*Campylobacter* may also be a good candidate marker genus in BD heifer rumens. This genus has been observed to cause higher risk of gastroenteritis for trekkers traveling from lowlands to high altitudes [39]. Intolerance of the high-altitude environment, particularly in BD, may be due to the increased abundance of this genus, which can also increase the incidence of other diseases [40]. Furthermore, some *Campylobacter* species might function as nitrate reducers in the rumen [41,42]. Nitrate is metabolized in blood and tissues to form nitric oxide (NO) and other bioactive nitrogen oxides [43,44]. NO is a vasodilator of pulmonary circulation [45], which relaxes vascular smooth muscle and plays a key role in decreasing pulmonary artery resistance and maintaining dilation of the pulmonary vasculature [46]. This finding is in line with our observations that *Campylobacter* was positively correlated to pulmonary pressure in heifers. In other words, increased abundance of *Campylobacter* in BD heifers may alter rumen nitrate metabolism and exacerbate altitude sensitivity via NO-mediated vasodilation. 

Genera *Anaerofustis* and *Catonella* were positively related to the mPAP. *Anaerofustis* is a gram-positive genus of the family *Eubacteriaceae* [47]. According to Kai et al., increased abundance of *Anaerofustis* might be associated with poor health (depression) in rats [48]. Although genus *Catonella* is poorly described in the rumen, it is a gram-negative bacterial genus from the family of *Lachnospiraceae* and has been associated with some oral disease [49,50]. Further study on the role of *Anaerofustis* and *Catonella* in bovine brisket disease is needed. Brisket disease risk in Holstein cows is heritable, but development of the disease is also related to non-genetic environmental factors [51,52]. These risk factors include age, diet, health status, and climate, all of which are also known to help shape the rumen microbial community. Observations of links between the microbial community, rumen metabolism, and BD warrant additional consideration. Moreover, our findings illustrated that rumen bacterial community and rumen metabolic changes were associated with bovine brisket disease. Further application of the key bacteria is worth considering to alleviate bovine brisket disease.

## 5. Conclusions

The development of the dairy industry in the plateau would benefit from the introduction of Holstein cows, but bovine brisket disease is an obstacle to their incorporation into the high-altitude environment. Findings from this study highlight significant alteration of Holstein heifer rumen bacterial communities and VFAs in bovine brisket disease. Genera *Ruminococcus* and *Treponema* significantly decreased in the rumen and were positively correlated with total VFA and acetic acid. Genus *Campylobacter* was associated with rumen VFA concentrations and was enriched in the rumens of heifers suffering from bovine brisket disease and may be a useful biomarker in future brisket disease work. This study provides an initial investigation of rumen bacterial community and rumen metabolic changes associated with bovine brisket disease. Further investigation of the role of key genera in bovine brisket disease is needed, with particular focus on candidate BD biomarkers in the rumen and potential BD mitigation strategies that leverage the rumen microbiome.

## Figures and Tables

**Table 1:** Mean pulmonary arterial pressure, blood oxygen saturation and average breathing rate of Holstein heifers.

Items	HH	BD	*p*-Value
mPAP (mmHg)	38.63 ± 1.56	74.73 ± 9.41	0.0018
Blood oxygen saturation (%)	88.33 ± 4.13	76.4 ± 4.75	0.0345
Average breathing rate (breaths/minute)	33.67 ± 6.08	17.2 ± 3.39	0.0114

mPAP = mean pulmonary arterial pressure; BD = brisket disease group; HH = healthy heifer group.

**Table 2:** Rumen volatile fatty acids (VFAs) of Holstein heifers.

Items	HH	BD	*p*-Value
Total VFA (mmol/L)	120.8 ± 4.84	77.30 ± 6.23	0.0006
Acetic acid (%)	66.46 ± 0.6839	63.19 ± 1.450	0.0758
Propionic acid (%)	17.46 ± 0.4576	19.04 ± 2.372	0.5303
Butyric acid (%)	11.22 ± 0.5580	10.97 ± 1.141	0.8458
Isobutyric acid (%)	1.483 ± 0.0509	1.560 ± 0.1287	0.5938
Valeric acid (%)	1.646 ± 0.0544	1.602 ± 0.1484	0.7891
Isovaleric acid (%)	1.720 ± 0.1434	1.943 ± 0.1151	0.2600

BD = brisket disease group; HH = healthy heifer group.

## Appendix A

**Table A1:** Ingredient and nutrient concentrations of experimental diets.

Item	Content
Ingredient/diet (g/100 g DM)	
Corn silage	36.40
Alfalfa hay	24.36
Oat hay	9.56
Corn grain	14.91
Soybean meal	4.47
Rapeseed meal	2.09
Cotton seed meal	1.79
Wheat bran	1.82
Cottonseed protein	1.49
Jujube powder	1.04
Premix	0.30
Limestone	0.50
Dicalcium phosphate	0.42
Sodium bicarbonate	0.75
Salt	0.24
Chemical composition (g/100 g DM)	
Crude protein	13.49
NE_L_ (MJ/kg)	6.02
Neutral detergent fiber	34.11
Acid detergent fiber	22.96
Ether extract	3.50
Ash	6.40
Calcium	0.73
Total phosphorus	0.47

Every kilogram of premix contained: vitamin A, 1,000,000 IU; Vitamin D, 65,000 IU; Vitamin E, 5000 IU; Fe, 2000 mg; Mn, 2550 mg; Zn, 5500 mg; Cu, 1750 mg; Co, 40 mg; I, 70 mg; and Se, 75 mg.

## References

[1] Pretty J.N. (1997). The sustainable intensification of agriculture. Nat. Resour. Forum.

[2] Hirschler V. (2016). Cardiometabolic risk factors in native populations living at high altitudes. Int. J. Clin. Pract..

[3] Krishnan G., Paul V., Hanah S., Bam J., Das P. (2016). Effects of climate change on yak production at high altitude. Indian J. Anim. Sci..

[4] Holt T.N., Callan R.J. (2007). Pulmonary arterial pressure testing for high mountain disease in cattle. Vet. Clin. N. Am..

[5] Malherbe C.R., Marquard J., Legg D.E., Cammack K.M., O’Toole D. (2012). Right ventricular hypertrophy with heart failure in Holstein heifers at elevation of 1,600 meters. J. Vet. Diagn. Investig..

[6] Heinonen I., Luotolahti M., Vuolteenaho O., Nikinmaa M., Saraste A., Hartiala J., Koskenvuo J., Knuuti J., Arjamaa O. (2014). Circulating N-terminal brain natriuretic peptide and cardiac function in response to acute systemic hypoxia in healthy humans. J. Transl. Med..

[7] Wang S., Azarfar A., Wang Y., Cao Z., Li S. (2018). N-carbamylglutamate restores nitric oxide synthesis and attenuates high altitude-induced pulmonary hypertension in Holstein heifers ascended to high altitude. J. Anim. Sci. Biotechnol..

[8] Russell J.B., Rychlik J.L. (2001). Factors that alter rumen microbial ecology. Science.

[9] Yohe T., Schramm H., White R., Hanigan M., Parsons C., Tucker H., Enger B., Hardy N., Daniels K. (2019). Form of calf diet and the rumen. II: Impact on volatile fatty acid absorption. J. Dairy Sci..

[10] Bond J., Donaldson A., Coumans J., Austin K., Ebert D., Wheeler D., Oddy V. (2019). Protein profiles of enzymatically isolated rumen epithelium in sheep fed a fibrous diet. J. Anim. Sci. Biotechnol..

[11] Roh S., Kuno M., Hishikawa D., Hong Y., Katoh K., Obara Y., Hidari H., Sasaki S. (2007). Identification of differentially expressed transcripts in bovine rumen and abomasum using a differential display method. J. Anim. Sci..

[12] Shabat S.K., Sasson G., Doron-Faigenboim A., Durman T., Yaacoby S., Berg Miller M.E., White B.A., Shterzer N., Mizrahi I. (2016). Specific microbiome-dependent mechanisms underlie the energy harvest efficiency of ruminants. ISME J..

[13] Poudel P., Froehlich K., Casper D.P., St-Pierre B. (2019). Feeding Essential Oils to Neonatal Holstein Dairy Calves Results in Increased Ruminal Prevotellaceae Abundance and Propionate Concentrations. Microorganisms.

[14] Tong J.J., Zhang H., Yang D.L., Zhang Y.H., Xiong B.H., Jiang L.S. (2018). Illumina sequencing analysis of the ruminal microbiota in high-yield and low-yield lactating dairy cows. PLoS ONE.

[15] Ishaq S.L., Yeoman C.J., Whitney T.R. (2017). Ground Juniperus pinchotii and urea in supplements fed to Rambouillet ewe lambs Part 2: Ewe lamb rumen microbial communities. J. Anim. Sci..

[16] Vlková E., Trojanová I., Rada V. (2006). Distribution of bifidobacteria in the gastrointestinal tract of calves. Folia Microbiol..

[17] Qiao G.H., Yu C.Q., Li J.H., Yang X., Zhu X.Q., Zhou X.H. (2013). Effect of high altitude on nutrient digestibility, rumen fermentation and basal metabolism rate in Chinese Holstein cows on the Tibetan plateau. Anim. Prod. Sci..

[18] Zhang F., Yang W., Deng Z., Wu W., Wu H., Chen J., Wang Y., Yang Y. (2010). Effect of glutamine on change of intestinal microecology in rats exposed to acute high altitude. Chin. J. Microbiol..

[19] Michaux H., Nichols S., Babkine M., Francoz D. (2014). Description of thoracoscopy and associated short-term cardiovascular and pulmonary effects in healthy cattle. Am. J. Vet. Res..

[20] Cao Z., Li S., Xing J., Ma M., Wang L. (2008). Effects of maize grain and lucerne particle size on ruminal fermentation, digestibility and performance of cows in midlactation. J. Anim. Physiol. Anim. Nutr..

[21] Sun W., Qian X., Gu J., Wang X.J., Zhang L., Guo A.Y. (2017). Mechanisms and effects of arsanilic acid on antibiotic resistance genes and microbial communities during pig manure digestion. Bioresour. Technol..

[22] Caporaso J.G., Lauber C.L., Walters W.A., Berg-Lyons D., Lozupone C.A., Turnbaugh P.J., Fierer N., Knight R. (2011). Global patterns of 16S rRNA diversity at a depth of millions of sequences per sample. Proc. Natl. Acad. Sci. USA.

[23] Xue D., Chen H., Luo X., Guan J., He Y., Zhao X. (2018). Microbial diversity in the rumen, reticulum, omasum, and abomasum of yak on a rapid fattening regime in an agro-pastoral transition zone. J. Microbiol..

[24] Zhou Q.Q., Yang D.Z., Luo Y.J., Li S.Z., Liu F.Y., Wang G.S. (2011). Over-starvation aggravates intestinal injury and promotes bacterial and endotoxin translocation under high-altitude hypoxic environment. World J. Gastroenterol..

[25] Gozho G., Mutsvangwa T. (2008). Influence of carbohydrate source on ruminal fermentation characteristics, performance, and microbial protein synthesis in dairy cows. J. Dairy Sci..

[26] Lei Y., Zhang K., Guo M., Li G., Li C., Li B., Yang Y., Chen Y., Wang X. (2018). Exploring the spatial-temporal microbiota of compound stomachs in a pre-weaned goat model. Front. Microbiol..

[27] Zhang J., Shi H., Wang Y., Li S., Cao Z., Ji S., He Y., Zhang H. (2017). Effect of dietary forage to concentrate ratios on dynamic profile changes and interactions of ruminal microbiota and metabolites in holstein heifers. Front. Microbiol..

[28] Hu R., Zou H., Wang Z., Cao B., Peng Q., Jing X., Wang Y., Shao Y., Pei Z., Zhang X. (2019). Nutritional interventions improved rumen functions and promoted compensatory growth of growth-retarded yaks as revealed by integrated transcripts and microbiome analyses. Front. Microbiol..

[29] Henderson G., Cox F., Ganesh S., Jonker A., Young W., Global Rumen Census C., Janssen P.H. (2015). Rumen microbial community composition varies with diet and host, but a core microbiome is found across a wide geographical range. Sci. Rep..

[30] Rosewarne C.P., Cheung J.L., Smith W.J., Evans P.N., Tomkins N.W., Denman S.E., Cuív P.Ó., Morrison M. (2012). Draft genome sequence of Treponema sp. strain JC4, a novel spirochete isolated from the bovine rumen. J. Bacteriol..

[31] Wang L.J., Zhang G.N., Xu H.J., Xin H.S., Zhang Y.G. (2019). Metagenomic analyses of microbial and carbohydrate-active enzymes in the rumen of holstein cows fed different forage-to-concentrate ratios. Front. Microbiol..

[32] Lim S., Seo J., Choi H., Yoon D., Nam J., Kim H., Cho S., Chang J. (2013). Metagenome analysis of protein domain collocation within cellulase genes of goat rumen microbes. Asian-Australas. J. Anim. Sci..

[33] Kala A., Kamra D., Kumar A., Agarwal N., Chaudhary L., Joshi C. (2017). Impact of levels of total digestible nutrients on microbiome, enzyme profile and degradation of feeds in buffalo rumen. PLoS ONE.

[34] Xu C., Sun R., Qiao X., Xu C., Shang X., Niu W. (2014). Protective effect of glutamine on intestinal injury and bacterial community in rats exposed to hypobaric hypoxia environment. World J. Gastroenterol..

[35] Zhou X., Jiang X., Yang C., Ma B., Lei C., Xu C., Zhang A., Yang X., Xiong Q., Zhang P. (2016). Cecal microbiota of Tibetan Chickens from five geographic regions were determined by 16S rRNA sequencing. Microbiology.

[36] Alvarez-Cilleros D., Ramos S., Lopez-Oliva M.E., Escriva F., Alvarez C., Fernandez-Millan E., Martin M.A. (2020). Cocoa diet modulates gut microbiota composition and improves intestinal health in Zucker diabetic rats. Food Res. Int..

[37] Moreno-Pérez D., Bressa C., Bailén M., Hamed-Bousdar S., Naclerio F., Carmona M., Pérez M., González-Soltero R., Montalvo-Lominchar M.G., Carabaña C. (2018). Effect of a protein supplement on the gut microbiota of endurance athletes: A randomized, controlled, double-blind pilot study. Nutrients.

[38] Barcenilla A., Pryde S.E., Martin J.C., Duncan S.H., Stewart C.S., Henderson C., Flint H.J. (2000). Phylogenetic relationships of butyrate-producing bacteria from the human gut. Appl. Environ. Microbiol..

[39] Khanna K., Mishra K.P., Ganju L., Kumar B., Singh S.B. (2018). High-altitude-induced alterations in gut-immune axis: A review. Int. Rev. Immunol..

[40] Yang B., Le J.Q., Wu P., Liu J.X., Guan L., Wang J.K. (2018). Alfalfa intervention alters rumen microbial community development in Hu lambs during early life. Front. Microbiol..

[41] Lin M., Guo W.S., Meng Q.X., Stevenson D.M., Weimer P.J., Schaefer D.M. (2013). Changes in rumen bacterial community composition in steers in response to dietary nitrate. Appl. Microbiol. Biotechnol..

[42] Zhao L.P., Meng Q.X., Ren L.P., Liu W., Zhang X.Z., Huo Y.L., Zhou Z.M. (2015). Effects of nitrate addition on rumen fermentation, bacterial biodiversity and abundance. Asian-Australas. J. Anim. Sci..

[43] Nyström T., Ortsäter H., Huang Z., Zhang F., Larsen F.J., Weitzberg E., Lundberg J.O., Sjöholm Å. (2012). Inorganic nitrite stimulates pancreatic islet blood flow and insulin secretion. Free Radic. Biol. Med..

[44] Wang R., Wang M., Ungerfeld E.M., Zhang X.M., Long D.L., Mao H.X., Deng J.P., Bannink A., Tan Z.L. (2018). Nitrate improves ammonia incorporation into rumen microbial protein in lactating dairy cows fed a low-protein diet. J. Dairy Sci..

[45] Fagan K.A., Morrissey B., Fouty B.W., Sato K., Harral J.W., Morris K.G., Hoedt-Miller M., Vidmar S., McMurtry I.F., Rodman D.M. (2001). Upregulation of nitric oxide synthase in mice with severe hypoxia-induced pulmonary hypertension. Respir. Res..

[46] Tonelli A.R., Haserodt S., Aytekin M., Dweik R.A. (2013). Nitric oxide deficiency in pulmonary hypertension: Pathobiology and implications for therapy. Pulm. Circ..

[47] Finegold S.M., Lawson P.A., Vaisanen M.L., Molitoris D.R., Song Y., Liu C., Collins M.D. (2004). Anaerofustis stercorihominis gen. nov., sp. nov., from human feces. Anaerobe.

[48] Zhang K., Fujita Y., Chang L.J., Qui Y.G., Pu Y.Y., Wang S.M., Shirayama Y., Hashimoto K. (2019). Abnormal composition of gut microbiota is associated with resilience versus susceptibility to inescapable electric stress. Transl. Psychiatry.

[49] Hernandez M., Planells P., Martinez E., Mira A., Carda-Dieguez M. (2020). Microbiology of molar-incisor hypomineralization lesions. A pilot study. J. Oral Microbiol..

[50] Lu H.F., Ren Z.G., Li A., Li J.Y., Xu S.Y., Zhang H., Jiang J.W., Yang J.Z., Luo Q.X., Zhou K. (2019). Tongue coating microbiome data distinguish patients with pancreatic head cancer from healthy controls. J. Oral Microbiol..

[51] Shirley K., Beckman D., Garrick D. (2008). Inheritance of pulmonary arterial pressure in Angus cattle and its correlation with growth. J. Anim. Sci..

[52] Anand I., Harris E., Ferrari R., Pearce P., Harris P. (1986). Pulmonary haemodynamics of the Yak, cattle, and cross breeds at high altitude. Thorax.

